# Prevalence and predictors of depression among women in Afghanistan: a cross-sectional study

**DOI:** 10.1007/s44202-023-00068-4

**Published:** 2023-03-06

**Authors:** Ahmad Neyazi, Ahmad Shoaib Haidarzada, Vanya Rangelova, Adiba Erfan, Bahara Bashiri, Mehrab Neyazi, Naweed Faizi, Hande Konşuk-Ünlü, Mark D. Griffiths

**Affiliations:** 1 Afghanistan Center for Epidemiological Studies, Herat, Afghanistan; 2 Mental Health Ward, Herat Regional Hospital, Herat, Afghanistan; 3 Department of Epidemiology and Disaster Medicine, Faculty of Public Health, Medical University Plovdiv, Plovdiv, Bulgaria; 4 Faculty of Medicine, Balkh University, Mazar-e-Sharif, Afghanistan; 5 Faculty of Pharmacy, Atefi Institute of Health Sciences, Herat, Afghanistan; 6 Faculty of Medicine, Afghan Swiss Higher Educational Institute, Kabul, Afghanistan; 7 Institute of Public Health, Hacettepe University, Ankara, Turkey; 8 Department of Psychology, Nottingham Trent University, Nottingham, UK

**Keywords:** Afghanistan, Depression assessment, Mental health, Women’s health

## Abstract

Depression is a very common mental health illness characterized by a cluster of signs and symptoms ranging from mood disturbances and sleep or appetite disorders to physical manifestations of the body. The purpose of the present study was to estimate the prevalence of depression and its related factors among Afghan women. A cross-sectional study was conducted among women between July 14, 2021, to August 15, 2021, in Kabul, Mazar-e-Sharif and Samangan provinces (Afghanistan). A total of 664 Afghan women participated in the study (mean age = 28.85 years; SD ± 11.57). In order to assess depression among participants, the 19-item validated Dari version of the Center for Epidemiological Studies–Depression Scale was used. Approximately four-fifths of the sample had depression symptoms (79.1%). Participants with depression symptoms were significantly more likely to (i) be aged over 30 years, (ii) live in rural areas, (iii) have a low-income level, (iv) not have an occupation, (v) have any disease/illness, and (vi) have experienced an event that had mentally affected them in the past month. Considering the high prevalence of depression symptoms among Afghan women, there is a need to integrate mental health services for women in Afghanistan. The use of interventions including regular screening for depression symptoms, and educating women and girls concerning the symptoms of depression for early self-diagnosis are essential.

**Supplementary Information:**

The online version contains supplementary material available at 10.1007/s44202-023-00068-4.

## Introduction

Depression is a very common mental health illness characterized by a cluster of signs and symptoms ranging from mood disturbances and sleep or appetite disorders to physical manifestations of the body [1, 2]. Based on a 2021 report by the World Health Organization (WHO), approximately 5% of adults worldwide suffer from depression [1] and it has been increasing over the past three decades [3]. Depression greatly affects the ability of the individual to perform well educationally and occupationally [2]. It has been proven that complex interplay between social, psychological, and biological factors are among the leading causes of depression [1]. Women are categorized as a high-risk group for poor mental health outcomes. Early childhood hardships, lack of educational activities, and poverty are all factors that can contribute to and/or hasten the development of depression [2].

COVID-19 has also increased prevalence of depression globally [4]. It led to 27.6% increase in major depressive disorders cases after 2020 [50]. COVID-19 caused depression mostly by decreasing mobility, increasing sickness (increase in daily SARS-CoV-2 infection rate), and an increase in mortality rate globally [5]. Other major factors that have impacted mental health and led to major depression include conflicts, migration, and displacement due to war, and the prevalence of lifetime major depressive disorder has been found to be 25% among adults living in low-income countries [6].

Afghanistan is a low-income country that has been facing socio-political unrest for decades. According to the Institute for Health Metrics and Evaluation, Global Burden of Disease, in 2019, 1.44 million individuals in Afghanistan (5.13%) suffered from depression [7]. After decades of conflicts with military interventions, the Afghan people have been facing an intergenerational mental health crisis resulting in chronic mental health illnesses including depression and post-traumatic stress disorder [8]. Research shows women are more prone to depression [9]. A recent national survey in Afghanistan reported that 47% of women suffered from mental health illnesses including depression [10]. An earlier survey conducted in 2003 evaluated the depression rate among women in Afghanistan. The survey reported high rates of clinical depression (73–78%) and suicidal ideation among Taliban-controlled regions versus those in a Pakistani refugee camp (28%) [11]. The provision of overall healthcare services including psychiatric services has been halted or inaccessible due to an ongoing political crisis in the country [12]. Moreover, the stigma attached to mental illness discourages individuals from seeking psychiatric help [8].

War has destroyed the local socio-cultural ecosystem in Afghanistan which has effects on multiple levels throughout the lives of the individuals. Women in Afghanistan face chronic trauma, emotional abuse, and patriarchal community ideals which may lead extreme depression and suicidal thoughts [13, 14]. Gender-based violence against women, forced marriages, and the impact of war are the most important factors for this [14]. Traditional practices and customs, early marriage, and teenage pregnancies make it more difficult for women to obtain an education, learn new skills, and inherit property, all of which contribute to poor mental health outcomes [15]. There are extremely few opportunities for women to become financially independent [16]. Extreme political unrest can cause poverty, food insecurity, and famine for women and children. Moreover, unreliable governmental-led mandates and a lack of access to legal representation further contribute to societal disparities and poor mental health outcomes [17].

The paucity of high-quality data on mental health problems and the lack of qualified human resources have hampered the development of cost-effective strategies and interventions to address the growing challenge of mental health in Afghanistan [18]. There is a scarcity of data concerning the mental health of women especially depression in Afghanistan in recent years. Only one recent study has examined depression among Afghan women (i.e., [10]) but it used non-culturally adopted scales and it acknowledged in the limitations that some of the questions used were not understandable by their participants. Also, to assess depression they used a scale that assessed post-traumatic stress disorder rather than one on depression specifically. Moreover, the data were collected in 2017 before the civil war. Therefore, the present study estimated the prevalence of depression and its related factors among Afghan women. The study focused on documenting the data based on different epidemiological variables, for example, age, economic status, occupation, and associated comorbidities. The study further examined depression (using a culturally appropriate scale to assess depression) in urban versus rural women of Afghanistan. It should also be noted that the data were collected during the civil war where many individuals were displaced from their homes in rural areas to urban areas of the country.

## Methods

### Participants and procedure

The present study comprised a cross-sectional study conducted among women between July 14, 2021, to August 15, 2021, in Kabul, Mazar-e-Sharif and Samangan provinces (Afghanistan). A print-based questionnaire was used to interview the participants. A non-probability-based convenience sampling method was used to recruit the participants in the study. More specifically, a total of 1000 women in the above-mentioned provinces were approached face-to-face and were asked to take part in the study, and a total of 664 women voluntarily participated (response rate = 66.4%). Women in the aforementioned cities who agreed to participate in the study answered the questions asked by the interviewers. Validated psychometric instruments were used in the questionnaire to assess the depression of participants. Providing verbal consent and being aged above 18 years were the pre-requisites for participation in the study.

### Measures

The survey used in the present study consisted of two sections: questions relating to socio-economic variables and questions screening for depression symptoms. The socio-demographic information section included questions on age, marital status, province, residency, education, economic status, occupation, cigarette smoking, presence of disease/illness, and whether any bad events had happened during the past month.

In order to assess depression among participants, the 19-item validated Dari version [19] of the Center for Epidemiological Studies–Depression (CES-D) Scale was used [20]. The scale comprises three main groups of items: negative items (e.g., *“I felt that I could not shake off the blues even with help from my family or friends”*), positive items (e.g., *“I felt hopeful about the future”*), and interpersonal relationship items (e.g., *“People were unfriendly”*). All the items are scored on a four-point scale from 0 (*“rarely or none of the time/less than one day during the past week”*) to 3 (“*Most of all of the time/5–7 days during the past week”*) and scores range from 0 to 60. The cut-off scores were: 0–15 considered as normal, and a score of 16 or over was considered to be as suffering from depression [20]. The Cronbach’s alpha in the present study was 0.87.

### Data analysis

Microsoft Excel 2016 was used for data entry and IBM SPSS version 26 (SPSS Inc., Chicago, IL, USA) was used for data analysis. Descriptive statistics were presented as means, standard deviations, frequencies and percentages of the socioeconomic variables and depression. A Kolmogorov–Smirnov test was used to assess the normality assumption of numeric variables. The differences between two independent groups with non-normal distributions were evaluated using Mann–Whitney U tests. When the assumption that the expected count less than 5 should not exceed 20% for variables was met, Pearson's chi-square was used to test to assess the association between categorical variables; if not, the exact chi-square test was used. If the statistically significant association was found after chi-square test, post-hoc comparisons with Bonferroni adjustment were performed. Multiple logistic regression analysis was used to report the predictors of depression. Hosmer–Lemeshow statistics were used to assess the goodness of fit the model. A *p*-value of less than 0.05 was considered significant in the present study although the significance levels were a lot lower following Bonferroni correction used in post hoc testing.

## Results

A total of 664 Afghan women participated in the study of which approximately four-fifths had depression symptoms (79.1%). The mean age of the participants in the study was 28.85 years (SD ± 11.57), ranging from 18 to 68 years. Almost two-thirds of the participants were young women aged between 18 to 29 years (66.7%). Almost half the participants were married (49.2%). Nine-tenths of participants were living in urban areas (90.4%). Approximately one-quarter of the participants were illiterate (23.8%). Only a small proportion of the participants had a (self-perceived) high income (15.7%). Approximately one-quarter of the participants had an occupation (26.4%). The presence of physical disease among Afghan women was 17.8% (Table 1).

**Table 1:** Characteristics of participants and their relationship with prevalence of depression

Characteristic	Category	N	(%)	Depression		*p*-value
				Yes, N (%)^a^	No, N (%)	
Age group	18–29 years	443	66.7	332 (74.9)	111 (25.1)^b^	< 0.001^b^*
	≥ 30 years	221	33.3	193 (87.3)	28 (12.7)^b^	
Marital status	Single	306	46.1	234 (76.5)	72 (23.5)	0.067^c^
	Married	327	49.2	262 (80.1)	65 (19.9)	
	Widow or divorced	31	4.7	29 (93.6)	2 (6.4)	
Province	Samangan	300	45.2	222 (74.0)	78 (26.0)	0.002^b,d*^
	Balkh	297	44.7	253 (85.2)	44 (14.8)	
	Kabul	67	10.1	50 (74.6)	17 (25.4)	
Residency	Urban	600	90.4	466 (77.7)	134 (22.3)	0.007^b^*
	Rural	64	9.6	59 (92.2)	5 (7.8)	
Education	Illiterate	158	23.8	144 (91.1)	14 (8.9)	< 0.001^b,e^*
	Primary school	55	8.3	49 (89.1)	6 (10.9)	
	Secondary school	101	15.2	87 (86.1)	14 (13.9)	
	High school	227	34.2	158 (69.6)	69 (30.4)	
	University	123	18.5	87 (70.7)	36 (29.3)	
Economic status	High income	104	15.7	96 (92.3)	8 (7.7)	< 0.001^2^,*
	Low income	560	84.3	429 (76.6)	131 (23.4)	
Occupation	Working	175	26.4	133 (76.0)	42 (24.0)	0.245^b^
	Non-working	489	73.6	392 (80.2)	97 (19.8)	
Cigarette smoking	Never smoked	657	98.9	519 (79.0)	138 (21.0)	1.000^c^
	Ex-smoker	5	0.8	4 (80.0)	1 (20.0)	
	Current smoker	2	0.3	2 (100.0)	0 (0.0)	
Presence of disease/illness	Yes	118	17.8	109 (92.4)	9 (7.6)	< 0.001^b^*
	No	546	82.2	416 (76.2)	130 (23.8)	
Experienced a bad event in the past month	Yes	456	68.7	380 (83.3)	76 (16.7)	< 0.001^b^*
	No	208	31.3	145 (69.7)	63 (30.3)	
Total		664	100.0	525 (79.1)	139 (20.9)	

*Statistically significant

^a^Row percentage

^b^Pearson chi-square

^c^Fisher’s Exact chi-square

^d^Post-hoc test with Bonferroni adjustment results: Balkh-Kabul *p* = 0.169; Balkh-Samangan *p* = 0.003; Kabul-Samangan *p* = 1.000

^e^Post-hoc test with Bonferroni adjustment results: Illiterate-Primary school *p* = 1.000; Illiterate-Secondary school *p* = 1.000; Illiterate-High school *p* < 0.001; Illiterate-University *p* < 0.001; Primary school-Secondary school *p* = 1.000; Primary school-High school *p* = 0.057; Primary school-University *p* = 0.133; Secondary school-High school *p* = 0.0235; Secondary school-University *p* = 0.948; High school-University *p* = 1.000

The analysis indicated that in relation to those who reported depressive symptoms compared to those who did not, there were statistically significant differences found between age groups (*p* < 0.001), province (*p* = 0.002), residency (*p* = 0.007), education level (*p* < 0.001), economic status (*p* < 0.001), presence of disease/illness (*p* < 0.001) and experiencing a bad event in the past month (*p* < 0.001). These findings indicate that the level of depression was greater among (i) older individuals, (ii) high income individuals, (iii) individuals who lived in rural areas. (iv) individuals with an illness/disease, and (v) individuals who had experienced a bad event in the past month (Table 1).

Binary logistic regression analysis was used to report the predictors of depression comprising the following variables: age group, residency, economic status, occupational status, presence of disease/illness, and experiencing a bad event in the past month. The model’s accuracy rate was 79.1%, which means 79.1% of the observations were correctly classified by the multiple logistic regression model. All the variables except for occupational status, significantly contributed to the regression model. Participants who were over 30 years old, who were living in rural areas, had low income level, did not have an occupation, had any disease/illness and experiencing a bad event in the past month were more likely to have depression symptoms (Table 2).

**Table 2:** Multiple logistic regression analysis of correlates of depression

Variable	Odds ratio (95%CI)	*p*-value
Age group (18–29 years [ref] vs > 30)	1.904 (1.185–3.059)	0.008*
Residency (urban [ref] vs rural)	3.099 (1.190–8.069)	0.021*
Economic status (high income[ref] vs low income)	2.620 (1.214–5.655)	0.014*
Occupation (yes [ref] vs no)	1.093 (0.708–1.687)	0.689
Presence of disease/illness (no [ref] vs yes)	2.284 (1.088–4.795)	0.029*
Experienced a bad event during the past month (no [ref] vs yes)	1.742 (1.161 2.613)	0.007*

*Statistically significant

The mean total CES-D score among 664 participating Afghan women with depression was 25.23 (SD ± 10.96). Women with depression in the age group of 30 years and above had higher negative affect depression score (21.95 ± 9.17 vs 18.20 ± 9.68, *p* < 0.001) and a higher total score (29.17 ± 10.90 vs 25.23 ± 10.96, *p* < 0.001) than those aged 18 to 29 years. However, there was no difference in the scores on the positive affect and interpersonal problems depression subscales by age (Table 3).

**Table 3:** Scores on the three factors of CES-D Scale among female adult subgroups by age

Factors		Age group		*p*-value
		18–29 years (n = 443)	≥ 30 years (n = 221)	
Negative items	Mean ± SD	18.20 ± 9.68	21.95 ± 9.17	< 0.001^a^*
	Median (Q_1_-Q_3_)	17.23 (9.69–26.92)	22.62 (15.08–29.08)	
Positive items	Mean ± SD	6.33 ± 3.08	6.23 ± 2.56	0.714^a^
	Median (Q_1_-Q_3_)	6.00 (4.00–9.00)	6.00 (5.00–8.00)	
Interpersonal relationship items	Mean ± SD	1.40 ± 1.52	1.57 ± 1.58	0.172^a^
	Median (Q_1_-Q_3_)	1.00 (0.00–2.00)	1.00 (0.00–3.00)	
Total score	Mean ± SD	25.23 ± 10.96	29.17 ± 10.90	< 0.001^a^*
	Median (Q_1_-Q_3_)	24.21 (15.79–21.05)	29.47 (21.05–37.89)	

*Q1* 1st quartile, *Q3* 3rd quartile

*Statistically significant

^a^Mann–Whitney test

## Discussion

In the present study, a very high level of depression symptoms among the studied women was found (i.e., 79.1%). It was estimated that depression was more prevalent among women above 30 years of age, living in rural areas, and having low income. Depressive disorders are common mental health disorders, occurring as early as three years of age and across all world regions [21]. Its symptoms among children can last longer than two weeks in children [22]. According to the Global Burden of Disease Study 2019, depressive and anxiety disorders are the most common mental health illnesses, for which very few people ever seek formal treatment [23]. Different studies have estimated consistently higher prevalence of depressive disorders among females than in males [24, 25]. The gender difference appears to be similar for high-income countries as it is in low-income and middle-income countries [26] although variations exist between regions [27]. A similar study done in Kashmir where there was ongoing low-level conflict reported 51% depression among women [28]. Mumford [29] reported 66% of women in two rural areas of Pakistan to be suffering from depression whereas Ahmad et al. [30] in another rural sample in Pakistan found that 72% of women were experiencing depression. A number of potential factors have been found that may contribute to higher rates of depression in a rural setting, including lack of access to jobs leading to unemployment [31], stigma associated with seeking treatment for mental illnesses [32], and poorer physical health [33]. All these results including the present study are significantly higher than the overall estimate of the depression prevalence in the community which ranges from 2.4% to 29.7% based on a meta-analysis from 30 different countries [34].

Afghanistan is a country torn apart by war conflicts for more than four decades. This inevitably has resulted in destruction of the economic, social, and cultural infrastructure of the country [10]. In recent years it has been acknowledged that war exacerbates the violence against women [13]. The lack of adequate psychiatric services due to the ongoing armed conflicts and the exposure of Afghan women to severe traumatic experiences and interpersonal violence have negatively affected their mental health. Additionally, women in Afghanistan are subject to patriarchal attitudes, they often do not have access to education and this reduces their opportunities for financial independence. The results from the present study correspond to previous Afghan studies on depression. In one study among 799 adults, it was estimated that 67.7% of respondents had experienced depressive symptoms. In the present study the prevalence of depressive symptoms among women was even higher—73.41% [35]. Scholte et al. conducted a study among 1011 individuals living in Nangarhar province in Afghanistan and it was estimated that 58.4% of women included in the study exhibited depressive symptoms [36].

The studies regarding depression among women have shown that increasing age among women is significantly associated with depression [29, 37]. A Pakistan study reported a 1.8% increase in the prevalence of depression with every one-year increase in age [38]. The present study supported this finding and was shown to be a significant factor in the multiple logistic regression analysis. As age increases, interpersonal stressors for women become greater and they are more susceptible to such stressors [39, 40].

Among adult samples, data from the Netherlands Mental Health Survey and Incidence Study showed that paid work, including full-time employment, was associated with a decreased prevalence of depressive and anxiety disorders for men and women, but in women, the protective role of working was restricted to those without children [41]. This might be explained by the additional responsibilities that women with children who have to take care of their children. These responsibilities can lead to females not having a job which can impact their social relationships. This concurs with the results from the present study in which depression was more prevalent among non-working women, although this variable was non-significant in the multiple logistic regression analysis. These findings suggest that the beneficial effect of employment on mental health among women is reduced if there is role overload due to multiple roles [42].

Marital status may be another factor contributing to the possibility of depression occurrence. In the present study, there was a high prevalence of depression among married and widowed women (80.1% and 93.6% respectively) compared to single women. In community surveys, married individuals have reported higher levels of depression than unmarried individuals and this association is stronger for women [43, 44]. This might be due to the fact that married women are more strongly exposed to traditional sex-role experiences than unmarried women [44]. Another possible explanation for the higher prevalence of depression among married women is the probable relationship dissatisfaction also due to traditional sex-role experiences. Although the present study did not specifically address the issue of reduced marital dissatisfaction, it has been linked to higher rates of current depression symptoms as well as a higher chance of developing future depressive symptoms. However, several other studies have reported higher depression prevalence among women who were unmarried, separated or divorced [45, 46].

Studies have shown an increased depression prevalence among those with low educational status [47, 48]. In the present study, the highest level of depression was among illiterate women (91.1%). This is consistent with findings from other studies in which it has been reported that higher levels of education can enhance female autonomy and confidence and is associated with a lower prevalence of depression [26, 49]. Having higher education level can have a protective role when it comes to depression. Education enhances coping mechanisms in multiple ways, increasing women's self-efficacy and subsequently self-esteem. Additionally, it provides women a greater sense of control over their environment and reduces their sense of helplessness in challenging circumstances [50].

The presence of an illness can also lower the quality of life an individual and can be a contributing factor to a depressive disorder. In the present study in the multiple logistic regression analysis, the presence of an illness proved to be statistically significant, and this is consistent with findings from other studies [51, 52].

There are a number of limitations regarding this study. The study’s sample size was relatively small to claim a country level study on mental health. Moreover, the study only included women living in urban areas of these provinces so was not necessarily representative of women in rural areas or in other regions of Afghanistan. Also, due to the cross-sectional design of the study, the causality between depression and other variables could not be determined.

### Recommendations

Considering the high prevalence of depression among women, the use of interventions including regular screening for depression symptoms, and educating women and girls on symptoms of depression for early self-diagnosis are essential and should be provided by the international and national health agencies active in the country. Also, due to the high prevalence of depression, critical planning for providing psychiatric treatment for elderly women, women living in rural areas, and women with diseases is necessary. Future studies should consider including people living in rural areas as well as focus on the causes of depression arising from the rules and laws being implemented by the Taliban. It is recommended that future studies examine other diseases and mental health conditions as there are many symptoms shared between mental health disorders.

### Conclusion

The results of the present study highlight the need to integrate mental health services for women in Afghanistan. Factors such as age group, residency, education, economic status, presence of disease/illness, and experiencing a bad event in the past month were significantly associated with presence of depressive symptoms.

## Supplementary Information

Below is the link to the electronic supplementary material.

Supplementary file1 (XLSX 167 KB)

## Data Availability

The datasets during and/or analyzed during the current study are available on request from the corresponding author.
